# Glucanocellulosic ethanol: the undiscovered biofuel potential in energy crops and marine biomass

**DOI:** 10.1038/srep13722

**Published:** 2015-09-01

**Authors:** Christian Falter, Claudia Zwikowics, Dennis Eggert, Antje Blümke, Marcel Naumann, Kerstin Wolff, Dorothea Ellinger, Rudolph Reimer, Christian A. Voigt

**Affiliations:** 1 Phytopathology and Biochemistry, Biocenter Klein Flottbek, University of Hamburg, Hamburg, Germany; 2 Heinrich Pette Institute, Leibniz Institute for Experimental Virology, Hamburg, Germany; 3 Max Planck Institute for the Structure and Dynamics of Matter, Hamburg, Germany

## Abstract

Converting biomass to biofuels is a key strategy in substituting fossil fuels to mitigate climate change. Conventional strategies to convert lignocellulosic biomass to ethanol address the fermentation of cellulose-derived glucose. Here we used super-resolution fluorescence microscopy to uncover the nanoscale structure of cell walls in the energy crops maize and Miscanthus where the typical polymer cellulose forms an unconventional layered architecture with the atypical (1, 3)-*β*-glucan polymer callose. This raised the question about an unused potential of (1, 3)-*β*-glucan in the fermentation of lignocellulosic biomass. Engineering biomass conversion for optimized (1, 3)-*β*-glucan utilization, we increased the ethanol yield from both energy crops. The generation of transgenic Miscanthus lines with an elevated (1, 3)-*β*-glucan content further increased ethanol yield providing a new strategy in energy crop breeding. Applying the (1, 3)-*β*-glucan-optimized conversion method on marine biomass from brown macroalgae with a naturally high (1, 3)-*β*-glucan content, we not only substantially increased ethanol yield but also demonstrated an effective co-fermentation of plant and marine biomass. This opens new perspectives in combining different kinds of feedstock for sustainable and efficient biofuel production, especially in coastal regions.

An increasing worldwide demand for energy combined with decreasing fossil energy resources not only fosters climate change^1^ but also explorations for fossil energy in sensitive ecosystems^2^. A key strategy to mitigate climate change is to substitute fossil by renewable energy sources. Because liquid fuels play a predominant role in the transportation sector, second generation biofuels from lignocellulosic feedstock reveal a high potential in substituting fossil fuels^3^. Restrictions in the production of ethanol from biomass mainly derive from the plant cell wall’s recalcitrance, which is primarily determined by cellulose crystallinity but also lignin and hemicellulose content^4^,^5^.

To improve second generation ethanol production, we tried to identify and increase the content of cell wall polymers that are easily degradable and contain readily fermentable residues. Hence, polymers that only consist of glucose would represent an optimal substrate for ethanol fermentation with efficient microorganisms like yeast (*Saccharomyces cerevisiae*). Apart from the major (1, 4)-*β*-glucan cell wall polymer cellulose^6^, only two other cell wall polymers consist entirely of glucose: (1, 3)-*β*-glucan, known as callose in plants^7^, and (1, 3;1, 4)-*β*-glucan, a mixed-linkage glucan found in plants of the order Poales, in horsetail (*Equisetum* spp.), and in bryophytes^8^. (1, 3)-*β*-glucan is important to maintain the vascular system, for pollen development, and cell plate formation in growing tissue as well as for defense responses^7^. Mixed-linkage glucan can serve as an energy storage and has a growth-related function in vegetative tissues of grasses^9^. Because of their biological function, the abundance of these *β*-glucan polymers has been considered low in lignocellulosic biomass^10^. Therefore, these polymers have not been targeted to improve saccharification. To test whether an undiscovered potential of *β*-glucan processing to ethanol would exist, we determine the (1, 3)-*β*-glucan and (1, 3;1, 4)-*β*-glucan content in lignocellulosic biomass of crops representing a major source of lignocellulosic feedstock from agriculture in temperate climates: barley (*Hordeum vulgare*), wheat (*Triticum aestivum*), and maize (*Zea mays*); as well as in the model plants Arabidopsis (*Arabidopsis thaliana*) and Brachypodium (*Brachypodium distachyon*), and the emerging, perennial grass Miscanthus (*Miscanthus* x *giganteus*). This low-input energy crop with high biomass yields in temperate climates has been proposed for sustainable lignocellulosic feedstock production^11^.

Whereas the (1, 3;1, 4)-*β*-glucan content was relatively low in all tested plant species (0.2–0.5%), the (1, 3)-*β*-glucan content was exceptionally high in maize and Miscanthus, reaching 2% and 5% of total dry leaf biomass, respectively (Fig. 1a). The specificity of the florescent dye aniline blue for staining (1, 3)-*β*-glucan rather than (1, 3;1, 4)-*β*-glucan allowed its usage in assays for (1, 3)-*β*-glucan quantification and microscopic localization (Supplementary Fig. 1). Confocal laser scanning microscopy (CLSM) revealed an unexpectedly high (1, 3)-*β*-content in epidermal leaf cells of maize and Miscanthus (Fig. 1b), which we did not observe in the other examined plant species (Supplementary Fig. 2). Moreover, localization microscopy (LM), which we recently established for super-resolution analysis of *β*-glucan polymer networks in plant cells^12^, facilitated three-dimensional rendering of (1, 3)-*β*-glucan and (1, 4)-*β*-glucan macrofibrils in cell walls (Fig. 1b). This microscopic technique revealed a parallel orientation of *β*-glucan polymer layers. The generation of videos based on three-dimensional rendered glucan macrofibrils data specifically addressed the visualization of direct interactions between the two cell wall polymers. Whereas in maize, interaction between (1, 3)-*β*-glucan and (1, 4)-*β*-glucan was mainly based on direct macrofibrils attachment (Supplementary Video 1), (1, 3)-*β*-glucan macrofibrils additionally partially surrounded (1, 4)-*β*-glucan macrofibrils in Miscanthus (Supplementary Video 2). This suggests the establishment of a tight polymer network that we also identified in epidermal leaf cells of Arabidopsis at sites of attempted pathogen penetration^12^. Our results from maize and Miscanthus provide first evidence that (1, 3)-*β*-glucan can be a major polymer of unchallenged secondary cell walls outside the vascular tissue. Interestingly, we also found a relatively high resistance of (1, 3)-*β*-glucan to chemical degradation by diluted trifluoroacetic and sulfuric acid (Supplementary Fig. 3), which supported the idea of an independent cell wall component rather than being part of the hemicellulose fraction^10^.

Before engineering an improved utilization of (1, 3)-*β*-glucan-enriched biomass, we developed an equation to estimate the increase in biomass saccharification after optimized (1, 3)-*β*-glucan hydrolysis:


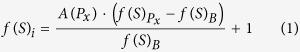


where *A*(*P*_*x*_) describes the relative amount of the glucan polymer *P*_*x*_, *f* (*s*) the glucose saccharification factor of the biomass *B* before optimization and of the glucan polymer *P*_*x*_ after optimized hydrolysis, and *f* (*s*)_*i*_the increase in glucose saccharification after optimization. Based on equation (1), we expected an improved saccharification only if *f* (*s*)_*Px*_ > *f* (*s*)_B_.

We analyzed the maize leaf biomass broth after dilute-sulfuric acid pretreatment, hydrolysis with the cell wall-degrading enzyme cocktail Accellerase 1500, and subsequent fermentation with a non-adapted, laboratory yeast strain. Here, we detected relative high contents of laminaribiose and –triose (Fig. 2a), which we distinguished from putative glucotrioses deriving from possible (1, 3;1, 4)-*β*-glucan degradation using a refractive index detector coupled to an HPLC-system (Supplementary Fig. 4). Due to their chemical composition and their relatively small size, we considered these (1, 3)-*β*-glucan degradation products as a potential, unused glucose source for fermentation. Therefore, we initiated experiments for optimizing (1, 3)-*β*-glucan hydrolysis and usage of (1, 3)-*β*-glucan degradation products for ethanol production. In a first step, we changed the yeast strain during fermentation to increase laminaribiose and –triose utilization during fermentation. The application of the yeast strain CEN.PK113-13D (CEN) that has been used to develop strains for optimized biomass fermentation^13^ significantly improved laminaribiose utilization during fermentation, but did not effected laminaritriose utilization (Fig. 2a). The heterologous expression of the bacterial laminaribiose ABC transporter (LBT) from *Clostridium thermocellum*^14^ in the yeast strain CEN (Supplementary Fig. 5a,b) further improved the laminaribiose and laminaritriose utilization (Fig. 2b), resulting in only residual amounts of laminaribiose and –triose in the fermentation broth (Fig. 2a). However, the utilization of laminaritetraose, long-chained (1, 3)-*β*-glucan, or oligomers deriving from (1, 4)-*β*-glucan and (1, 3;1, 4)-*β*-glucan hydrolysis was not facilitated (Supplementary Fig. 5c). Because of the efficient utilization of these two (1, 3)-*β*-glucan oligomers by CEN+LBT, we considered laminaribiose and –triose as direct contributors to the overall glucose saccharification. Hence, the generation of this yeast strain represented a decisive step in engineering optimized (1, 3)-*β*-glucan utilization from *β*-glucan-enriched biomass.

To further improve saccharification of (1, 3)-*β*-glucan-rich biomass, we initially screened 38 (1, 3)-*β*-glucanases from bacteria, fungi, and plants (Supplementary Table 1) that we heterologously expressed and purified from *Escherichia coli.* Six (1, 3)-*β*-glucanases showed a higher efficiency in (1, 3)-*β*-glucan hydrolysis than the Accellerase enzyme cocktail and a commercially available (1, 3)-*β*-glucanase from *Trichoderma reesei*, a well-studied and widely used fungus in second generation biofuel production^15^ (Supplementary Fig. 6a). After confirming expression of these six (1, 3)-*β*-glucanases in *E. coli* (Supplementary Fig. 7), we determined their optimal pH and temperature range for enzymatic activity (Supplementary Fig. 8). Under optimal conditions for each enzyme, we identified highest (1, 3)-*β*-glucanase activity for the enzyme from *Flavobacterium johnsoniae* (Supplementary Table 1), which was 3.3-times higher than the enzymatic activity of the commercially available (1, 3)-*β*-glucanase from *T. reesei*, resulting in an almost 70% hydrolyzing efficiency (Supplementary Fig. 9a). Combining the saccharification efficiency from non-optimized biomass processing (Fig. 2a) and the (1, 3)-*β*-glucan content of maize and Miscanthus biomass (Fig. 1a), we predicted a 4.0% increase in saccharification efficiency for maize and a 9.3% increase for Miscanthus using equation (1). The experimental data only slightly deviated from our prediction showing a 5.3% increase for maize and a 9.0% increase for Miscanthus (Fig. 2c). The improved second generation ethanol production reflected the efficiency of engineered (1, 3)-*β*-glucan processing due to i) optimization of its enzymatic hydrolysis and ii) enhanced utilization of its degradation products by the engineered yeast strain CEN+LBT, resulting in an increased ethanol production of 5.7% for maize and 14.4% for Miscanthus (Fig. 2d).

Our results from saccharification and fermentation of maize and Miscanthus biomass revealed a direct correlation between the (1, 3)-*β*-glucan content of the feedstock and an increased ethanol yield. Hence, we concluded that a further (1, 3)-*β*-glucan enrichment in biomass would result in increased ethanol yields. To test this hypothesis, we followed two strategies: i) increasing the (1, 3)-*β*-glucan content in potential feedstock for sustainable biomass production using a biotechnological approach; and ii) identifying new sources of (1, 3)-*β*-glucan-enriched biomass that could be used in our adapted fermentation process.

To increase the (1, 3)-*β*-glucan content in feedstock for sustainable biomass production, we overexpressed the GFP-tagged (1, 3)-*β*-glucan synthase gene *PMR4* (*POWDERY MILDEW RESISTANT4*) from Arabidopsis in Miscanthus (line *35S:PMR4-GFP*). *PMR4* overexpression in Arabidopsis increased (1, 3)-*β*-glucan content at infection sites but not in unchallenged tissue^16^. In contrast, we observed a constitutive increase in (1, 3)-*β*-glucan content in *35S:PMR4-GFP* Miscanthus lines, which was proportional to the relative *PMR4* expression level and reached a maximum of 8.5% in leaf tissue (Supplementary Fig. 10a,b). This result suggests different regulatory mechanisms of (1, 3)-*β*-glucan biosynthesis in Miscanthus and Arabidopsis, which would also explain the strong differences in their overall (1, 3)-*β*-glucan content (Fig. 1). As expected from Arabidopsis^16^, PMR4-GFP was localized at the plasma membrane whereas single GFP of a transgenic Miscanthus control line was detectable in cytosolic strands (Supplementary Fig. 10c). We predicted an increase in saccharification efficiency of about 16% in the *35S:PMR4-GFP* line with the highest (1, 3)-*β*-glucan content of 8.5% after optimized hydrolysis, which was relatively close to our experimental results showing a saccharification increase of 14.5%. (Fig. 3b). The improved saccharification of this Miscanthus line resulted in an increase in ethanol production of 20% compared to non-optimized Miscanthus wild-type biomass processing (Fig. 3c). These results revealed a previously undiscovered potential in the layered architecture of maize and Miscanthus leaf cell walls that contain an atypically high content of (1, 3)-*β*-glucan, which was unleashed by engineering optimized enzymatic hydrolysis and yeast fermentation. Moreover, (1, 3)-*β*-glucan enrichment represents a new target in breeding energy crops for improved second generation ethanol production.

In our second approach to identify new sources of (1, 3)-*β*-glucan-enriched biomass, we considered brown macroalgae with a naturally high (1, 3)-*β*-glucan content as a putative source. To examine the potential of (1, 3)-*β*-glucan utilization for ethanol production from this marine feedstock, we collected thalli of the brown macroalgae bladderwrack (*Fucus vesiculosus*) from the German Baltic Sea shore at Eckernförde near Hamburg (Fig. 3d). We determined an astonishing (1, 3)-*β*-glucan content of 15.3% (Fig. 3a) confirming previous studies of this macroalga^17^. CLSM revealed (1, 3)-*β*-glucan deposition in all tissues of the blade (Fig. 3e). However, highest (1, 3)-*β*-glucan accumulation occurred within epidermal and cortex cells (Fig. 3f). The generation of three-dimensional videos from (1, 3)-*β*-glucan accumulation within the bladderwrack tissue allowed us to distinguish between different deposition patterns. Whereas elongated cells of the central pith region revealed a scattered (1, 3)-*β*-glucan deposition pattern (Supplementary Video 3), a relatively compact layer of (1, 3)-*β*-glucan was intracellular deposited in cortex and especially epidermal cells (Supplementary Video 4). Based on our previous results, we hypothesized that the optimized processing of (1, 3)-*β*-glucan-enriched bladderwrack biomass would result in increased ethanol production. We first tested the six (1, 3)-*β*-glucanases with highest activity on brown algae-derived (1, 3)-*β*-glucan as substrate. Similar to our previous test, the enzyme from *F. johnsoniae* showed highest activity with a hydrolyzing efficiency of 37.5% (Supplementary Fig. 9b). Using this (1, 3)-*β*-glucanase in addition to the Accellerase enzyme cocktail for biomass hydrolysis after dilute-sulfuric acid pretreatment, we increased bladderwrack biomass saccharification by 45.6% (Fig. 3g), which was close to the prediction of 46.4% using equation (1). Consequently, the ethanol yield was 50.5% higher in optimized CEN+LBT-driven fermentation compared to non-optimized (1, 3)-*β*-glucan processing (Fig. 3h).

Similar to Miscanthus, brown macroalgae have been considered for sustainable biomass production^18^, however, without competing for arable land and food production. Because we demonstrated optimized ethanol production for both, (1, 3)-*β*-glucan-enriched plant and marine feedstock, we proposed our engineered biomass processing for co-fermentation of Miscanthus and bladderwrack biomass (Fig. 4a). A successful co-fermentation using equal amounts of Miscanthus and bladderwrack biomass proved the applicability of this engineered production approach (Fig. 4b). Regarding saccharification of mixed Miscanthus and bladderwrack biomass, we identified enzymatic hydrolysis as a field of further improvement. Here, the release of laminaribiose from (1, 3)-*β*-glucan was specifically inhibited during mixed Miscanthus and bladderwrack biomass processing compared to single biomass processing whereas inhibition did not occur for laminaritriose or glucose release in the mixed biomass approach (Fig. 4b).

Because brown macroalgae do not contain lignin and (1, 3)-*β*-glucan substantially contributed to improve second generation ethanol production in our study, we considered the produced ethanol as glucanocellulosic.

The effective co-fermentation opens new perspectives for sustainable and efficient ethanol production in bio-refineries, especially in coastal regions that combine the potential of offshore macroalgae aquaculture and proximate energy crop cultivation. An example for a coastal region that would fulfill these prerequisites is Schleswig-Holstein in northern Germany. Existing and planned offshore wind parks in the North and Baltic Sea would facilitate effective macroalgae aquacultures^19^, and a high potential for Miscanthus cultivation in Schleswig-Holstein was shown in our recent study^20^. Hence, these coastal regions would represent prototypic sites for future biorefineries for plant and marine biomass co-fermentation, combining short delivery distances of feedstock with a high abundance of renewable electricity for processing, which could help to promote large-scale energy transition projects like the ambitious German *Energiewende*^21^.

## Methods

### Biological material

Brachypodium (*Brachypodium distachyon*, inbred line Bd 21^22^), barley (*Hordeum vulgare*, cultivar Golden Promise^23^), wheat (*Triticum aestivum*, cultivar Nandu, Lochow-Petkus, Bergen-Wohlde, Germany), maize (*Zea mays*, inbred line A188^24^), and Miscanthus (*Miscanthus x giganteus*) were cultivated as described in Meineke *et al.*^25^. Arabidopsis (*Arabidopsis thaliana*, wild-type Columbia) was cultivated as described in Stein *et al.*^26^. Naturally dried leaf material was harvested manually at its final developmental stage after senescence and 2 additional weeks of drying^25^. Biomass was additionally dried at 50 °C for 2 days in a drying oven. Washed ashore thalli of the brown macroalga bladderwrack (*Fucus vesiculosus*) were collected in November from the Baltic Sea shore at Eckernförde (Schleswig-Holstein, Germany, geographical position: 54 °27'57.5″N 9°50'28.0″E) and dried at 50 °C for 3 days. Plant and alga biomass was homogenized with a mill fitted with a 0.5 mm mesh screen prior processing. Material subject to (1, 3)-*β*-glucan extraction was ground in liquid nitrogen using a mortar and pestle.

### (1, 3)-*β*-Glucan extraction and determination

20 mg of mortared and lyophilized leaf or alga biomass was destained in ethanol (96%) at 50 °C and 600 rpm for 10 min. Subsequent procedures of (1, 3)-*β*-glucan determination followed the description in Voigt *et al.*^27^. Ethanol was removed after centrifugation (2 min, 10,000 g), and the sample was dried using a centrifugal evaporator. After a washing step with H_2_O, the sample was dried again in a centrifugal evaporator. For (1, 3)-*β*-glucan extraction, the sample was resuspended in 400 μl of 1 M NaOH and incubated at 80 °C and 600 rpm for 1 h. After centrifugation (10 min, 2000 g), the supernatant was used for the aniline blue fluorescence assay for (1, 3)-*β*-glucan determination. 5 μl sample were mixed with 45 ml H_2_O, 5 μl HCl (1 M), 220 μl K_2_HPO_4_ (150 mM), and 5 μl aniline blue fluorochrome (ABF, 0.1 mg·ml^−1^ in H_2_O, Biosupplies, Australia). Standards ranging from 0 to 20 μg ml^−1^ were generated from purified (1, 3)-*β*-glucan from *Euglena gracilis* (Sigma-Aldrich, Germany) in the same way as described for plant and alga samples. Additional standards were generated accordingly from oat and barley (1, 3;1, 4)-*β*-glucan deriving from the mixed-linkage beta-glucan kit (Megazyme, Ireland) to verify the specificity of ABF in staining (1, 3)-*β*-glucan. Fluorescence measurement was performed in 96-well plates with the microplate reader Synergy HT (BioTek, USA; absorbance filter: 380/20 nm, emission filter: 460/40 nm).

### (1, 3;1, 4)-*β*-Glucan determination

Mixed linkage (1, 3;1, 4)-*β*-glucan in plant biomass was determined according to the manufacturer’s description of the mixed-linkage beta-glucan kit (Megazyme). An additional 5 ml H_2_O was added to the samples (750 mg milled leaf biomass) in the initial incubation step in a water bath (100°C). Calculation of the (1, 3;1, 4)-*β*-glucan content followed the instructions of the manufacturer’s manual.

### Confocal laser-scanning microscopy

The confocal laser-scanning microscope Zeiss LSM 780 (Carl Zeiss MicroImaging GmbH, Germany) was used for the localization of pontamine fast scarlet 4B (S4B, Sigma-Aldrich)-stained (1, 4)-*β*-glucan in leaf samples and ABF-stained (1, 3)-*β*-glucan in leaf and bladderwrack samples. To verify the specificity of ABF in staining (1, 3)-*β*-glucan, (1, 3)-*β*-glucan from *E. gracilis* (Sigma-Aldrich) as well as (1, 3;1, 4)-*β*-glucan from oat and barley (Megazyme) was suspended in H_2_O and stained with ABF and used in CLSM. In addition, the green fluorescence protein (GFP)-tagged (1, 3)*-β*-glucan synthase PMR4 and single GFP in leaves of transformed Miscanthus lines was localized by CLSM. For cross sections of leaves, samples were embedded in 9% Agarose, and sections were made with a vibratome (Carl Zeiss MicroImaging). The setup for CLSM analysis of stained cell wall polymers and GFP followed the description in Ellinger *et al.*^16^ and Eggert *et al.*^12^.

### Super-resolution microscopy

A custom modified Nikon stochastic optical reconstruction microscope (N-STORM, Nikon GmbH, Germany) was used to analyze the (1, 3)-*β*-glucan/(1, 4)-*β*-glucan polymer network of ABF and S4B stained maize and Miscanthus cross sections. The microscopic setup and image reconstruction was done according to Eggert *et al.*^12^.

### Treatment and fermentation of plant biomass

For pretreatment, 5 g of milled leaf biomass was mixed with 43 ml sulfuric acid (1.75% (v/v)) and autoclaved for 15 min at 120 °C. Subsequent procedures of enzymatic hydrolysis and fermentation followed the description in Meineke *et al.*^25^. To test the impact of the (1, 3)-*β*-glucanase from *F. johnsoniae*, 500 ng of the purified enzyme were additionally added to fermentation reactors and incubated for 24 h and 200 rpm at 37 °C. Fermentations were initiated with the inoculation of 2 ml of overnight yeast cultures of the non-adapted, laboratory strain MaV203 (MaV, Life Technologies), CEN, or CEN+LBT (generated in this study). Amounts of glucose, laminaribiose, laminaritriose, and ethanol in fermentation supernatants were quantified with a refractive index detector on an ICS-5000 system (Dionex, USA) with a HPX 87H column (Bio-Rad, USA, mobile phase 0.005 M H_2_SO_4_, flow rate 0.6 ml·min^−1^, column temperature: 50 °C, refractive index detector) as described in Meineke *et al.*^25^. In addition to laminaribiose and laminaritriose, the trisaccharides glucotriose (I) (*β*-D-Glc-(1 → 3)-*β*-D-Glc-(1 → 4)-D-Glc), glucotriose (II) (*β*-D-Glc-(1 → 4)-*β*-D-Glc-(1 → 3)-D-Glc) were used as standards (all standard oligosaccharides from Megazyme) to distinguish between possible degradation products from (1, 3)-*β*-glucan and (1, 3;1, 4)-*β*-glucan.

### Cloning and Miscanthus transformation

We generated two vector constructs for the transformation of Miscanthus: i) overexpression of the callose synthase gene *PMR4* from Arabidopsis (At4g03550) fused to *GFP* and ii) overexpression of single *GFP*, both under control of the 35S promoter. *PMR4-GFP* and *GFP* were amplified from the vector pCAMBIA-35S:PMR4-GFP^16^ using primers in PCR reactions that provide DNA recombination sequences (*attB* sites) at their 5′ and 3′ ends (PMR4-5′attB: 5′-GGGGACAAGTTTGTACAAAAAAGCAGGCTATGAGCCTCCGCCACCGC, GFP-5′attB: 5′-GGGGACAAGTTTGTACAAAAAAGCAGGCTTGGAGATC CAAACAATGAGTAAAG, GFP-3′attB: 5′-GGGGACCACTTTGTACAAGAAAGCTGGG TTAAGCTTGAATTCTTATT TGTATA) for utilization with the Gateway cloning technology. *PMR4-GFP* and *GFP* were introduced into the plant expression vector pIPKb004^28^, which provided 35S promoter-driven gene expression. Generated vector constructs containing *35S:PMR4-GFP* and *35S:GFP* expression cassettes were transformed into Agrobacterium (*Agrobacterium tumefaciens*, strain GV3101). The generation of transgenic Miscanthus lines followed the principal procedure of Agrobacterium-mediated callus transformation and selection on hygromycin-containing plant cell culture medium. Resistance to hygromycin was provided by the used plant expression vector pIBKb004. A detailed description of the transformation procedure is provided in the Supplementary Information.

### Statistical analysis

Descriptive statistics including the mean and the standard error of the mean (SE) along with the Tukey range test for multiple comparison procedures in conjunction with an ANOVA were used to determine significant differences. *P* < 0.05 was considered significant.

## Additional Information

**How to cite this article**: Falter, C. *et al.* Glucanocellulosic ethanol: the undiscovered biofuel potential in energy crops and marine biomass. *Sci. Rep.* **5**, 13722; doi: 10.1038/srep13722 (2015).

## Supplementary Material

Supplementary Information

Supplementary Video 1

Supplementary Video 2

Supplementary Video 3

Supplementary Video 4

## Figures and Tables

**Figure 1:**
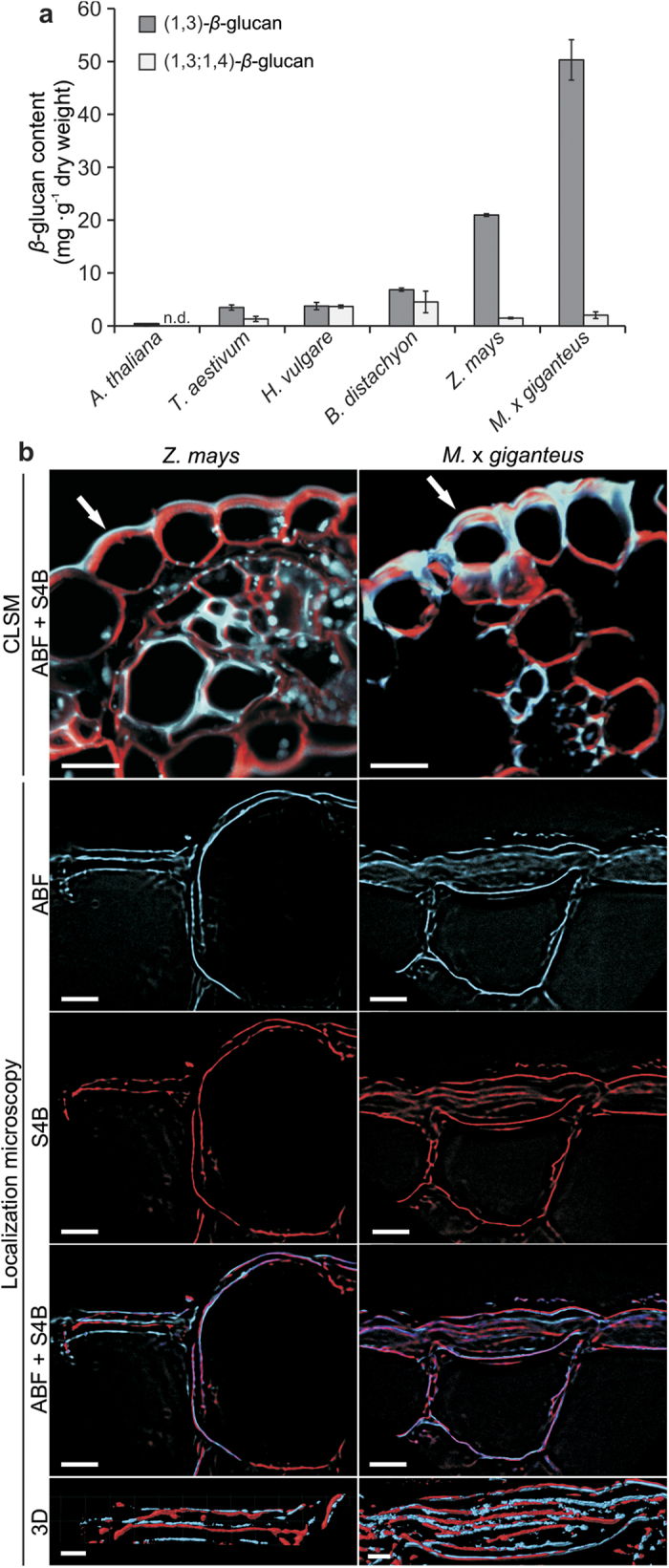
Layered cell wall architecture in maize and Miscanthus. (**a**) *β*-Glucan content in senesced leaf biomass. n.d., not detectable. Values represent the mean of three independent biological experiments. Error bars represent ± SE. (**b**) Confocal laser-scanning microscopy (CLSM) of aniline blue fluorochrome (ABF)-stained (1, 3)-*β*-glucan (blue channel) and pontamine fast scarlet 4B (S4B)-stained (1, 4)-*β*-glucan (red channel) cross sections of leaves from maize (*Z. mays*) amd Miscanthus (*M*. x *giganteus*). White arrows indicate epidermal cells with a high (1, 3)-*β*-glucan content and sites of localization microscopy (LM) application. 3D, surface rendering of *β*-glucan networks. Scale bars: CLSM, 50 μm; LM ABF/S4B, 5 μm; LM 3D, 2 μm.

**Figure 2:**
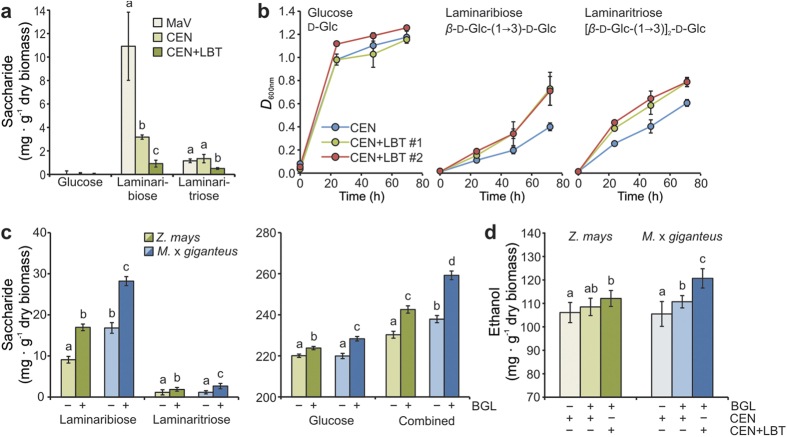
Engineered saccharification and ethanol production of (1, 3)-*β*-glucan-rich biomass. (**a**) Amounts of glucose and the oligosaccharides laminaribiose and –triose remaining in the fermentation broth after 48 h of maize leaf biomass fermentation comparing yeast strains MaV (non-adapted), CEN (adapted), and CEN+LBT (engineered). (**b**) *In vitro* growth assays of yeast strains CEN and CEN+LBT on substrates as indicated. (**c**) Amounts of laminaribiose and –triose (left panel) as well as glucose and the combined amount of these three (1, 3)-*β*-glucan hydrolysis products (right panel) as indicators for changes in saccharification efficiency of maize and Miscanthus leaf biomass due to additional *F. johnsoniae* (1, 3)*-β*-glucanase application (−/+ BGL) after standard biomass pretreatment. (**d**) Ethanol production after 48 h of fermentation using yeast strains CEN and CEN+LBT and −/+ BGL. Values represent the mean of three independent biological experiments. Letters a, b, c: groups with significant difference, *P* <  0.05 based on Tukey’s test. Error bars represent ± SE.

**Figure 3:**
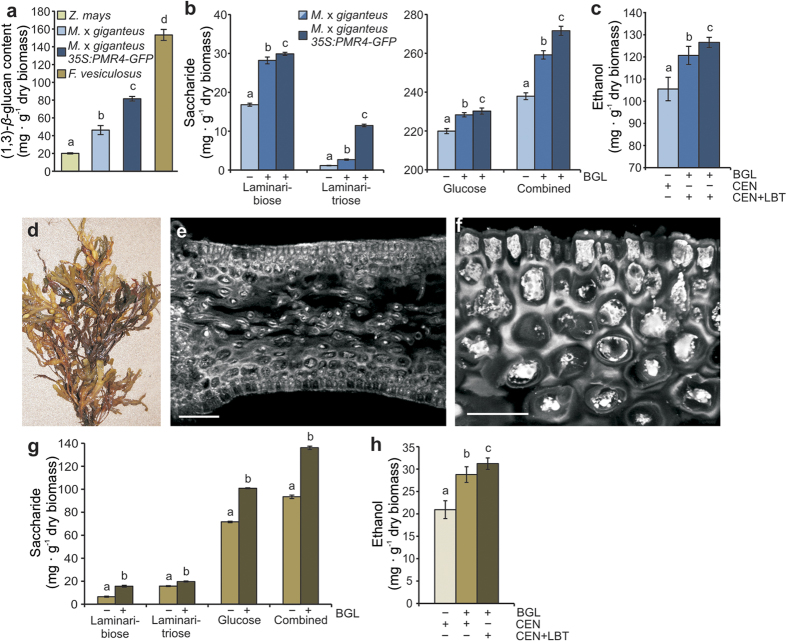
Enhanced saccharification and ethanol production in (1, 3)-*β*-glucan-enriched Miscanthus and marine biomass. (**a**) (1, 3)-*β*-glucan content in engineered Miscanthus leaf (*35S:PMR4-GFP*) and bladderwrack (*F. vesiculosus*) biomass. (**b**) Amounts of the oligosaccharides laminaribiose and –triose (left panel) as well as glucose and the combined amount of these three (1, 3)-*β*-glucan hydrolysis products (right panel) as indicators for changes in saccharification efficiency of Miscanthus wild-type and engineered leaf biomass due to additional *F. johnsoniae* (1, 3)*-β*-glucanase application (−/+ BGL) after standard biomass pretreatment. (**c**) Ethanol production after 48 h of fermentation using yeast strains CEN (adapted) and CEN+LBT (engineered) and −/+ BGL. (**d**) Thallus morphology of bladderwrack. Photo courtesy of Christian A. Voigt. (**e**) Micrograph showing cross section of the bladderwrack’s blade after aniline blue fluorochrome-staining of (1, 3)*-β*-glucan. Micrograph taken by confocal laser-scanning microscopy. Scale bar, 100 μm. (**f**) Magnification of the blade’s epidermal and cortex cells as indicated in (e). Scale bar = 20 μm. (**g**) Amounts of laminaribiose, –triose,glucose, and the combined amount of these three (1, 3)-*β*-glucan hydrolysis products as indicators for changes in saccharification efficiency of bladderwrack biomass −/+ BGL. (**h**) Ethanol production after 48 h of fermentation using the yeast strains CEN and CEN+LBT and −/+ BGL. Values represent the mean of three independent biological experiments. Letters a, b, c: groups with significant difference, *P* < 0.05 based on Tukey’s test. Error bars represent ± SE.

**Figure 4:**
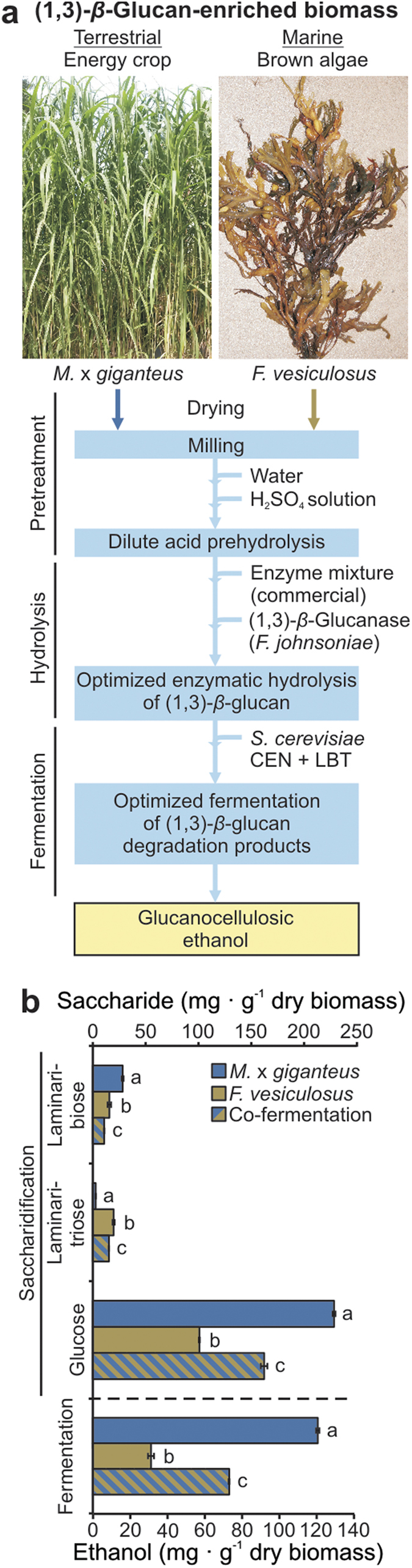
Co-fermentation of (1, 3)-*β*-glucan-enriched terrestrial and marine biomass. (**a**) Schematic overview of parallel biomass processing. Photos courtesy of Christian A. Voigt. (**b**) Saccharification efficiency and ethanol production under optimized co-processing of Miscanthus and bladderwrack biomass. Values represent the mean of two independent biological experiments. Letters a, b, c: groups with significant difference, *P* < 0.05 based on Tukey’s test. Error bars represent ± SE.
