# Supplementary material for: Glucanocellulosic ethanol: the undiscovered biofuel potential in energy crops and marine biomass
Source: Sci Rep. 2015 Sep 1;5:13722. doi: 10.1038/srep13722 (PMC4555182; doi:10.1038/srep13722)
Supplement: Supplementary Information [file srep13722-s1.pdf]

SUPPLEMENTARY INFORMATION

Glucanocellulosic ethanol: the undiscovered biofuel potential in energy crops and marine biomass

Authors: Christian Falter[1]*, Claudia Zwikowics[1]*, Dennis Eggert[2,3], Antje Blümke[1], Marcel Naumann[1], Kerstin Wolff[1], Dorothea Ellinger[1], Rudolph Reimer[2], Christian A. Voigt[1]†

Affiliations:
[1]Phytopathology and Biochemistry, Biocenter Klein Flottbek, University of Hamburg, Hamburg, Germany.
[2]Heinrich Pette Institute, Leibniz Institute for Experimental Virology, Hamburg, Germany.
[3]Max Planck Institute for the Structure and Dynamics of Matter, Hamburg, Germany.
*These authors contributed equally to this work.
†Correspondence to: E-mail: christian.voigt@uni-hamburg.de (C.A.V.)

Content:

Supplementary Figures

Fig. 1: Specificity of aniline blue in (1,3)-β-glucan determination and microscopic localization. (p.1)
Fig. 2: Cell wall architecture of leaf cells. (p.2)
Fig. 3: Chemical hydrolysis of *β*-glucans. (p.3)
Fig. 4: Chromatograms of *β*-glucan di- and trimers using an RI detector. (p.4)
Fig. 5: Characterization of laminaribiose transporter-expressing yeast strains. (p.5)
Fig. 6: (1,3)-*β*-Glucanase screening. (p.6)
Fig. 7: Verification of (1,3)-*β*-glucanase purification and protein size. (p.8)
Fig. 8: Characterization of enzymatic activity of (1,3)-*β*-glucanases. (p.9)
Fig. 9: Enzymatic activity of (1,3)-*β*-glucanases under optimal conditions. (p.10)
Fig. 10: Characterization of transgenic Miscanthus lines. (p.11)

Supplementary Tables

Tab. 1: Origin of (1,3)-β-glucanase genes used for heterologous expression in *E. coli*. (p.12)
Tab. 2: DNA sequence of gene encoding the laminaribiose ABC transporter from *C. thermocellum* after codon usage optimization for yeast. (p.14)

Supplementary Video Legends (p.15)

Supplementary Methods (p.16)

SUPPLEMENTARY INFORMATION

Supplementary Figures

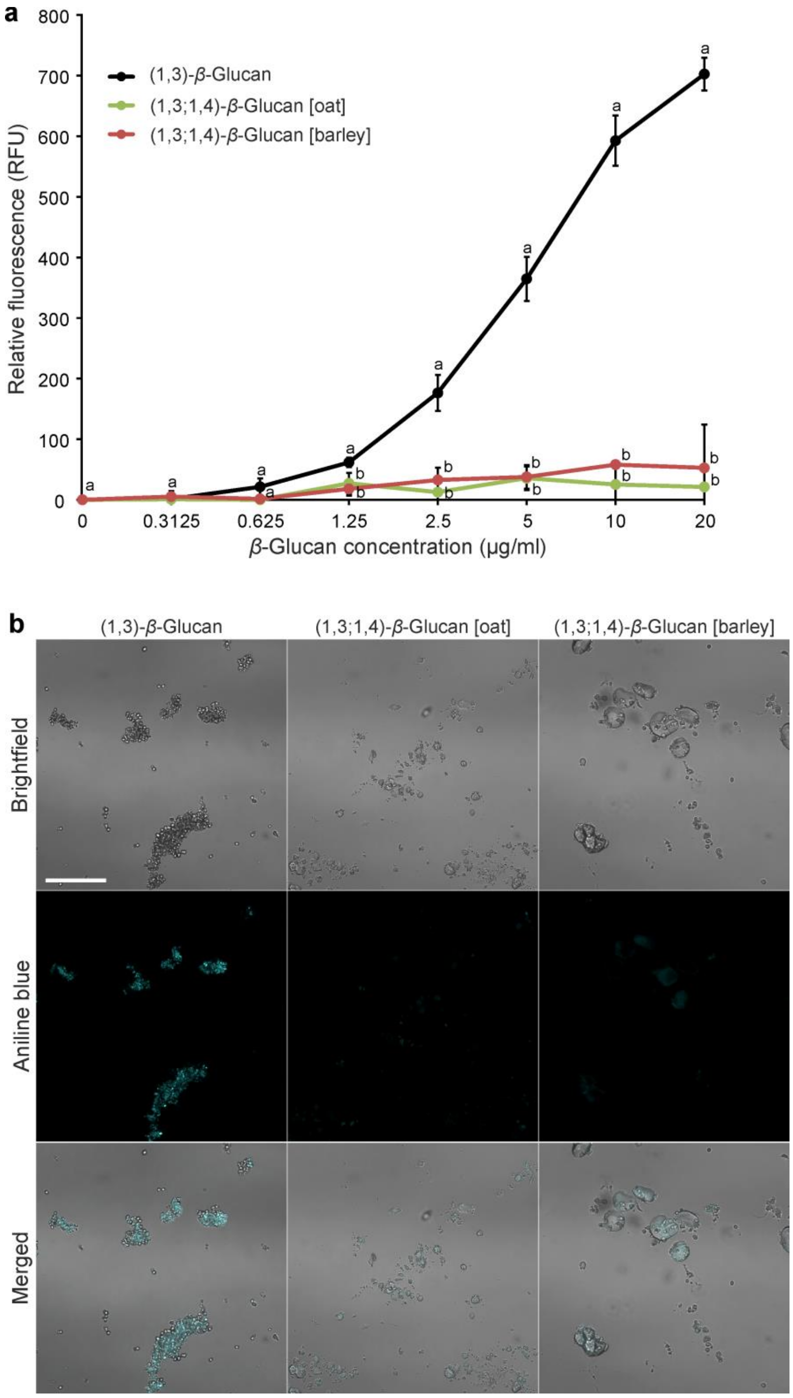

Supplementary Figure 1. Specificity of aniline blue in (1,3)-*β*-glucan determination and microscopic localization.

(1,3)-*β*-glucan from *E. gracilis* and (1,3;1,4)-*β*-glucan from oat and barley were used as reference polymers and stained with aniline blue fluorochrome (ABF). (**a**) Concentration-dependent ABF-emission of ABF-stained *β*-glucan polymers in fluorescence assays. Values represent the mean of two independent biological experiments. Letters a, b: groups with significant difference, $P < 0.05$ based on Tukey's test. Error bars represent ± SE. (**b**) Micrographs of ABF-stained *β*-glucan polymers suspended in water by confocal laser-scanning microscopy. Scale bar = 50 µm.

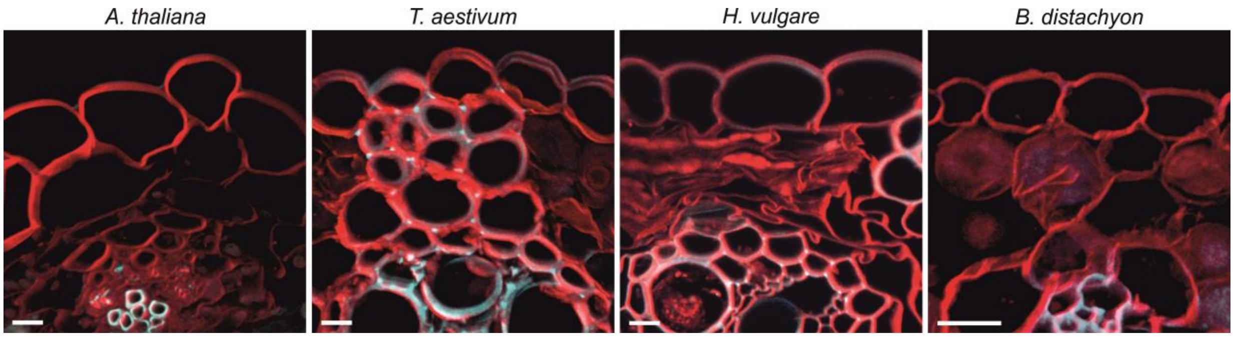

Supplementary Figure 2. Cell wall architecture of leaf cells.

Confocal laser-scanning microscopy of aniline blue fluorochrome-stained (1,3)-*β*-glucan (blue channel) and pontamine fast scarlet 4B -stained (1,4)-*β*-glucan (red channel) cross sections of leaves from the model plant Arabidopsis (*A. thaliana*), wheat (*T. aestivum*), barley (*H. vulgare*), and the model grass Brachyipodium (*B. distachyon*). Scale bars, 10 µm.

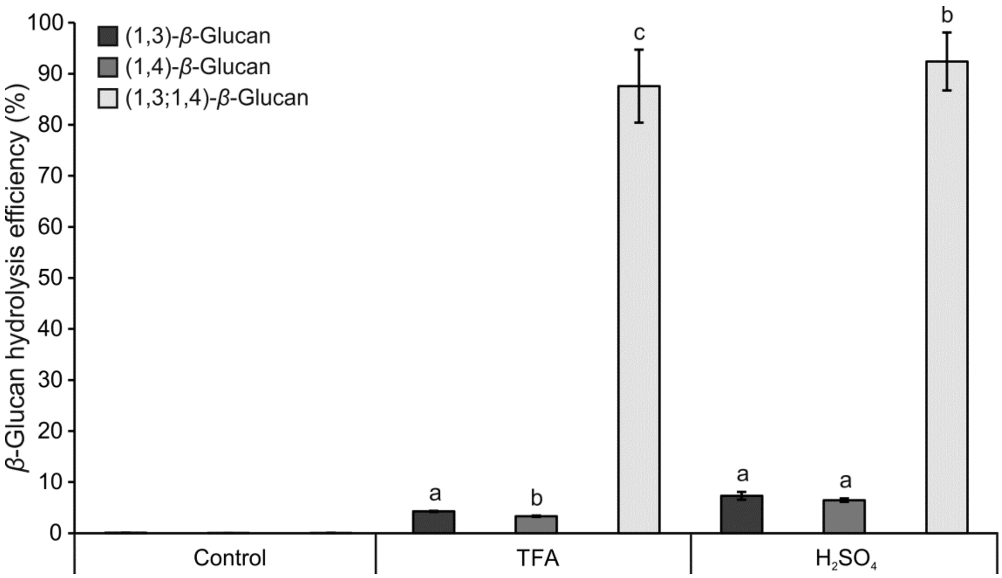

Supplementary Figure 3. Chemical hydrolysis of *β*-glucans.

β-Glucan polymers were treated with 1 M trifluoroacetic acid (TFA) and 1 M sulfuric acid (H_2SO_4) for 3 h at 105°C. Samples without TFA or H_2SO_4 treatment served as control. Amount of released glucose was used for calculation of *β*-glucan hydrolysis efficiency. Values represent the mean of two independent biological experiments. Letters a, b, c: groups with significant difference, $P < 0.05$ based on Tukey's test. Error bars represent ± SE.

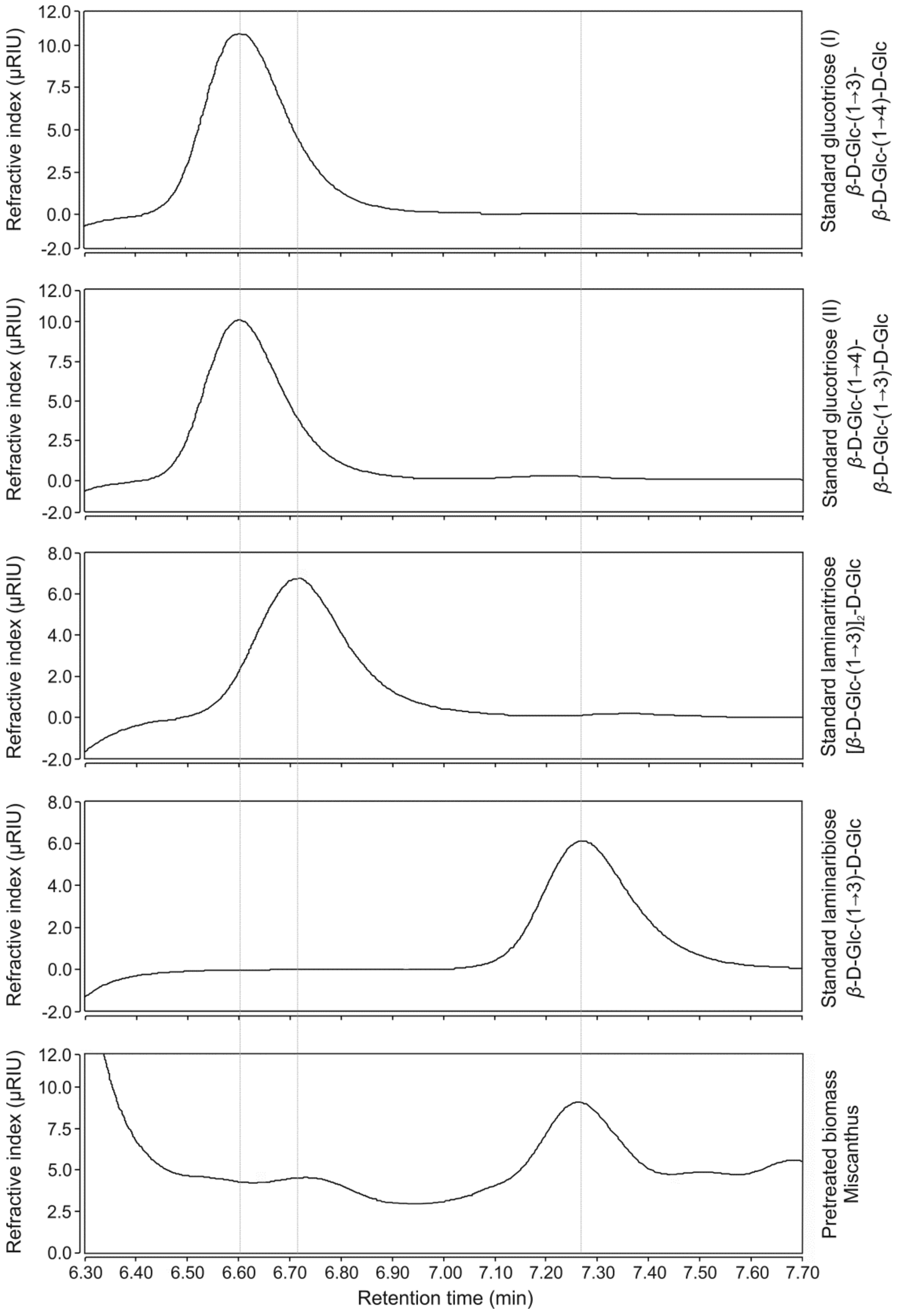

Supplementary Figure 4. Chromatograms of *β*-glucan di- and trimers using an RI detector.

Chromatograms of standard *β*-glucan di-and trimers (concentration: 0.4 µg·ml^{-2}) were obtained with an HPLC-coupled refractive index (RI) detector. Chromatogram of thermo-chemically and enzymatically pretreated Miscanthus biomass as an example for laminaribiose and –triose detection in biomass samples. Grey, dotted lines indicate retention times of (1,3;1,4)-*β*-glucan trimers (left), laminaritriose (middle), and laminaribiose (right). Two independent biological experiments gave similar results.

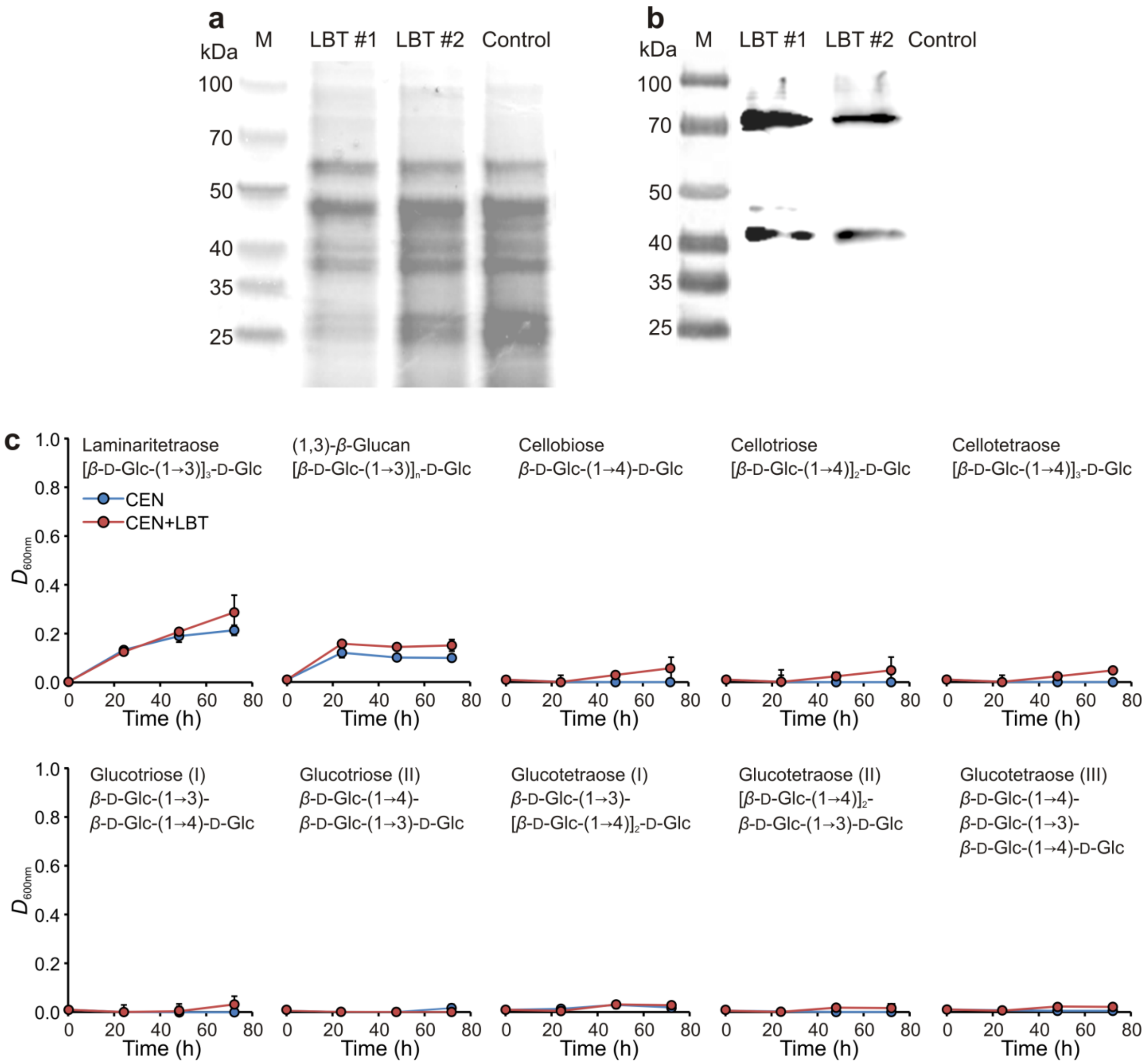

Supplementary Figure 5. Characterization of laminaribiose transporter-expressing yeast strains. Yeast strain CEN was transformed with yeast expression vector carrying bacterial laminaribiose ABC transporter (LBT) from *C. thermocellum*. Two independent clones (CEN+LBT #1 and #2) were randomly chosen for characterization. (**a**) Separation of total protein extracts on SDS-PAGE as loading control prior Western blot analysis. Proteins stained with Coomassie. Protein samples derived from of CEN+LBT strains #1 and #2 and control strain carrying the empty vector. M, Spectra Multicolor Broad Range Ladder (Thermo Scientific). (**b**) Western blot analysis of protein samples as indicated in (a) using a primary anti-His antibody. Expected size of LBT (incl. C-terminal His tag): 42 kDa. Additional hybridization of LBT-His at approx. 80 kDa indicated possible dimerization. (**c**) In vitro growth assays of the CEN and the CEN+LBT (strain #2) yeast strains on substrates as indicated. Values represent the mean of two independent biological experiments. No significant difference, $P < 0.05$ based on Tukey's test. Error bars represent ± SE.

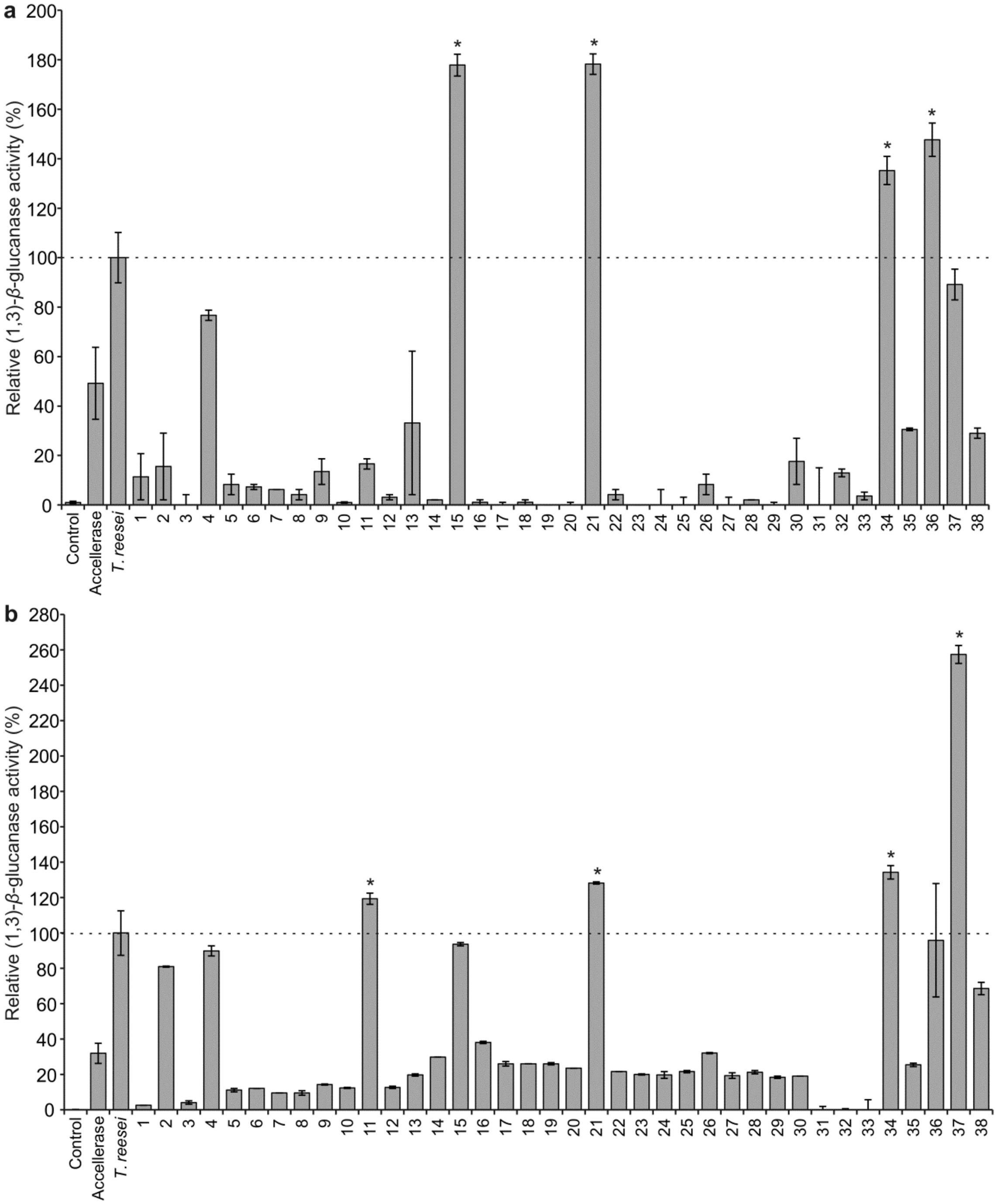

Supplementary Figure 6. (1,3)-*β*-Glucanase screening.

(1,3)-*β*-Glucanases (see supplementary table 1 for the sources of each of the numbered enzymes) were heterologously expressed und purified from *E. coli* via N-terminal His-tag fusion. Enzymatic activity of commercially available (1,3)-*β*-glucanase from *T. reesei* (Sigma-Aldrich) served as reference, Accellerase 1500 cellulose enzyme complex (Danisco) as additional hydrolysis control. (1,3)-*β*-glucan from *E. gracilis* (**a**) or *L. digitata* (**b**) were used as substrate in activity assays (conditions: pH 6, 37°C). (to be continued on next page)

Supplementary Figure 6. (1,3)-*β*-Glucanase screening. (continued)

Amount of released glucose was used for calculation of (1,3)-*β*-glucanase activity. Values represent the mean of two independent biological experiments. *Enzymatic activity significantly higher than reference activity from *T. reesei* (1,3)-*β*-glucanase at $P < 0.05$ based on Tukey's test. Error bars represent ± SE. (1,3)-*β*-glucanases #11, #15#, #21, #34, #36, and #37 were selected for further characterization due to highest enzymatic activity

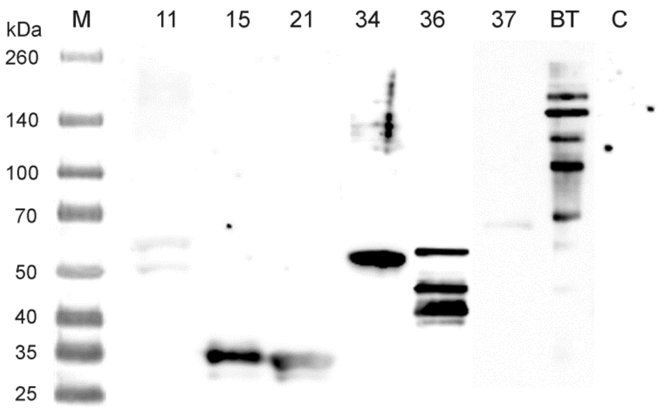

Supplementary Figure 7. Verification of (1,3)-*β*-glucanase purification and protein size.

Western blot analysis of purified (1,3)-*β*-glucanases showing higher enzymatic activity than the reference enzyme from *T. reesei* in (1,3)-*β*-glucanase screening (Supplementary Fig. 4) using an anti-His antibody. Protein extraction and purification from empty vector *E. coli* strain served as control (C). Expected protein sizes of tested (1,3)-*β*-glucanases (#11 - #37) can be found in supplementary table 1. M, Spectra Multicolor Broad Range Ladder (Thermo Scientific); BT, Biotinylated Protein Ladder (Cell Signaling Technology, USA).

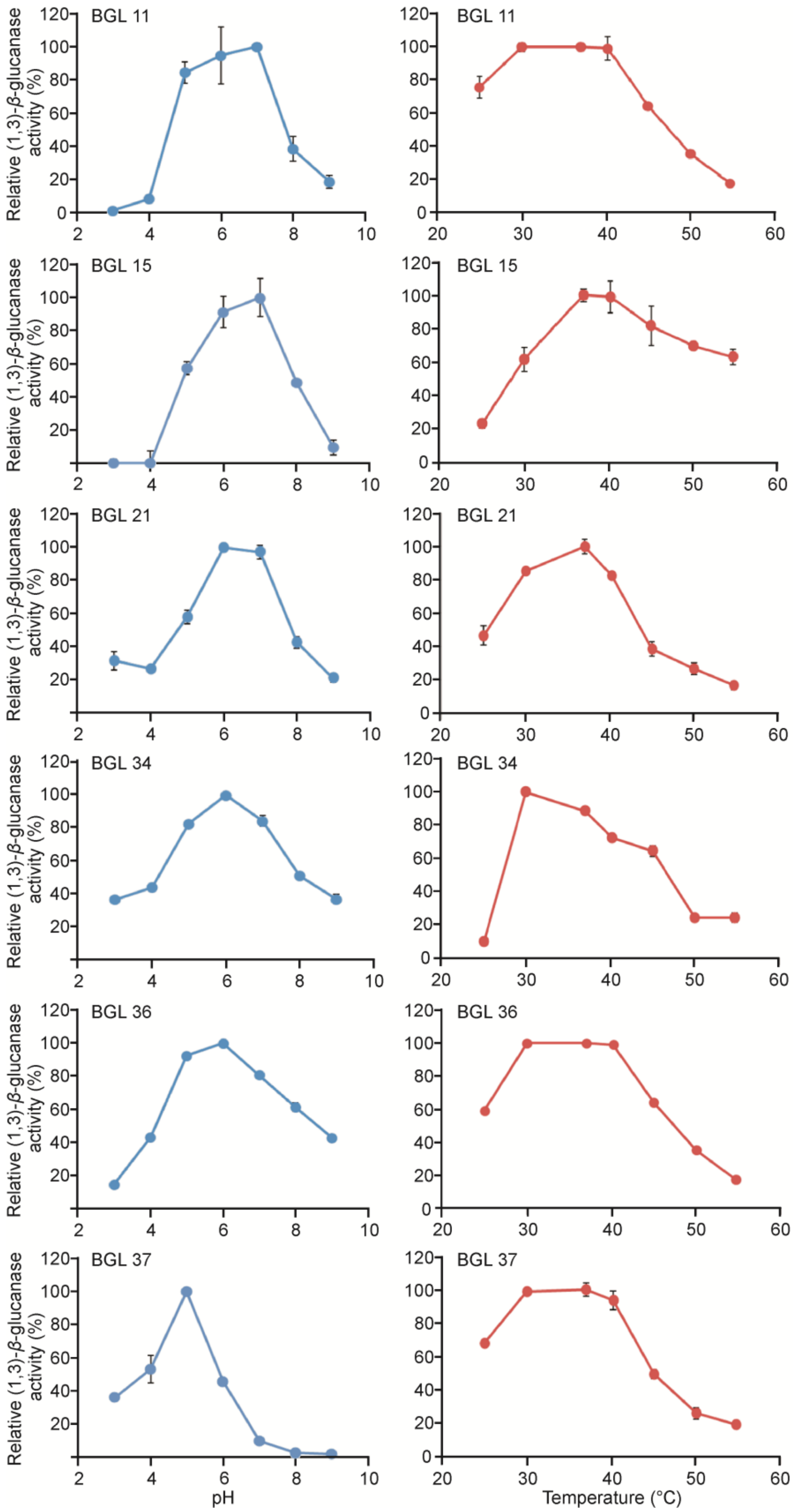

Supplementary Figure 8.

Characterization of enzymatic activity of (1,3)-*β*-glucanases.

Determination of optimal pH and temperature range of purified (1,3)-*β*-glucanases (BGL #11 – BGL #37, Supplementary Tab. 1). (1,3)-*β*-glucan from *E. gracilis* was used as substrate in activity assays under conditions as indicated. Amount of released glucose was used for calculation of (1,3)-*β*-glucanase activity. Values represent the mean of two independent biological experiments. Error bars represent ± SE.

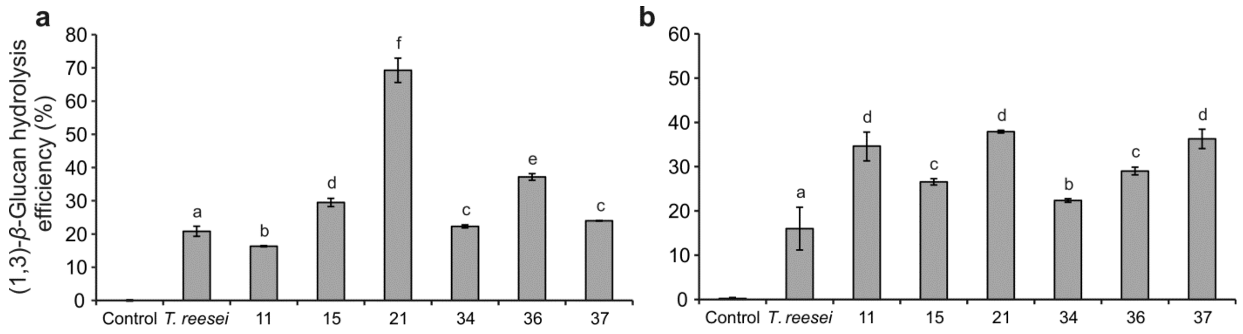

Supplementary Figure. 9. Enzymatic activity of (1,3)-*β*-glucanases under optimal conditions. Determination of enzymatic activity of purified (1,3)-*β*-glucanases (#11 - #37, Supplementary Tab. 1) under optimal enzymatic activity conditions (Supplementary Fig. 6) for each protein. Enzymatic activity of commercially available (1,3)-*β*-glucanase from *T. reesei* (Sigma-Aldrich) served as reference. (1,3)-*β*-glucan from *E. gracilis* (**a**) or *L. digitata* (**b**) was used as substrate in activity assays. Amount of released glucose was used for calculation of (1,3)-*β*-glucan hydrolysis efficiency of each enzyme. Values represent the mean of three independent biological experiments. Letters a, b, c, d, e, f: groups with significant difference, $P < 0.05$ based on Tukey's test. Error bars represent ± SE.

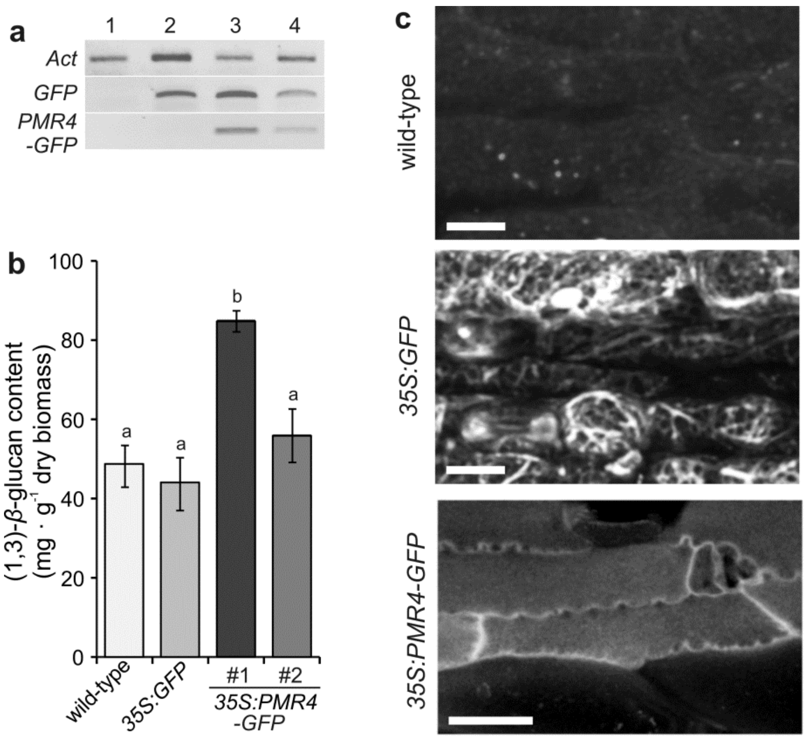

Supplementary Figure 10. Characterization of transgenic Miscanthus lines.

Agrobacterium-mediated transformation of Miscanthus (*M.* x *giganteus*) calli was used to generate transgenic Miscanthus lines. For increasing (1,3)-*β*-glucan content, the transgenic Miscanthus lines *35S:PMR4-GFP* #1 and #2 with an overexpression of the (1,3)-*β*-glucan synthase gene *PMR4* (*POWDERY MILDEW RESISTANT 4*) from Arabidopsis fused to the green fluorescence protein GFP were generated. A *GFP* overexpression line (*35S:GFP*) served as control. (**a**) *GFP* and *PMR-GFP* gene expression in leaf tissue of transgenic Miscanthus lines. Gene expression in non-transgenic wild-type plants was used as control. RNA was isolated from leaf tissue and used as template in complementary DNA generation. *Actin* (*Act*) gene expression served as reference. A repeat experiment gave similar results. Miscanthus lines: 1, wild-type; 2, *35S:GFP*; 3, *35S:PMR4-GFP* #1, 4, *35S:PMR4-GFP* #2. (**b**) Determination of (1,3)-*β*-glucan content in leaf tissue of Miscanthus wild-type and transgenic lines. Values represent the mean of three independent biological experiments. Letters a, b: groups with significant difference, $P < 0.05$ based on Tukey's test. Error bars represent ± SE. Based on the results of the (1,3)-*β*-glucan content, transgenic Miscanthus line *35S:PMR4-GFP* #1 was used in all subsequent experiments and referred to as "*35S:PMR4-GFP*". (**c**) Confocal laser-scanning microscopy to localize the fusion protein PMR4-GFP in the Miscanthus overexpression line *35S:PMR4-GFP* and single GFP in the control line *35S:GFP*. Micrographs display the 3D projection of epidermal leaf cells; shown is the view from the outside to the leaf surface. Micrographs are representative for the localization of single GFP in cytosolic strands in line *35S:GFP* and of PMR4-GFP at the plasma membrane in line *35S:PMR4-GFP* after examining at least 25 regions on 5 independent leaves from each line. Scale bars, 20 µm.

Supplementary Tables

Supplementary Table 1.

Origin of (1,3)-*β*-glucanase genes used for heterologous expression in *E. coli*. Enzyme size calculated on the basis of the amino acid sequence encoded by the respective (1,3)-*β*-glucanase gene.

No.	Species	Kingdom	Enzyme size [kDa]	GenBank
1	*Acremonium cellulolyticus* (*Talaromyces cellulolyticus*)	Fungi	95.8	BD168028.1
2	*Agaricus bisporus*	Fungi	50.7	XM_006454084.1
3	*Agaricus bisporus*	Fungi	19.5	X92961.1
4	*Aspergillus flavus*	Fungi	101.4	XM_002374364.1
5	*Aspergillus flavus*	Fungi	50.9	XM_002376609.1
6	*Aspergillus fumigatus* (strain Af293)	Fungi	43.6	XM_741253.2
7	*Aspergillus fumigatus* (strain Af293)	Fungi	86.5	XM_741429.1
8	*Aspergillus fumigatus* (strain Af293)	Fungi	111.1	XM_745080.1
9	*Aspergillus fumigatus* (strain Af293)	Fungi	94.1	XM_745984.1
10	*Aspergillus fumigatus* (strain Af293)	Fungi	100.9	XM_745719.1
11	*Aspergillus fumigatus* (strain Af293)	Fungi	49.5	XM_745017.1
12	*Aspergillus fumigatus* (strain Af293)	Fungi	95.8	XM_742417.1
13	*Aspergillus fumigatus* (strain Af293)	Fungi	90.6	XM_749766.1
14	*Bacillus circulans*	Bacteria	136.9	DI025015.1
15	*Bacillus clausii* (strain KSM-K16)	Bacteria	35.4	NC_006582.1
16	*Candida albicans* (strain SC5314)	Fungi	128.7	XM_716200.1
17	*Chaetomium thermophilum*	Fungi	70.3	DQ888228.1
18	*Chaetomium thermophilum*	Fungi	99.4	EF648280.1
19	*Clostridium thermocellum*	Bacteria	100.8	L04735.1
20	*Coccidioides immitis* (strain RS)	Bacteria	57.4	XM_001245402.1
21	*Flavobacterium johnsoniae* (strain UW101)	Bacteria	33.8	NC_009441.1
22	*Hypocrea lixii* (*Trichoderma harzianum*)	Fungi	85.9	CAA58889.1
23	*Hypocrea lixii* (*Trichoderma harzianum*)	Fungi	56.4	ACM42429.1
24	*Hypocrea lixii* (*Trichoderma harzianum*)	Fungi	52.5	FJ589724.1
25	*Lycopersicon esculentum* (tomato)	Plantae	41.0	X74906.1
26	*Lycopersicon esculentum* (tomato)	Plantae	41.0	X74905.1
27	*Neosartorya fischeri* (strain NRRL 181)	Fungi	38.3	NW_001509760.1
28	*Neurospora crassa* (strain OR74A)	Fungi	109.6	XM_952460.1
29	*Oryza sativa* (rice)	Plantae	25.5	NM_001066906.1
30	*Oryza sativa* (rice)	Plantae	56.1	NM_001057345.1

SupplementaryTable 1. (continued)

No.	Species	Kingdom	Enzyme size [kDa]	GenBank
31	*Oryza sativa* (rice)	Plantae	45.9	NM_001070740.1
32	*Stachybotrys echinata*	Fungi	31.4	AF435067.1
33	*Streptomyces avermitilis* (strain MA-4680)	Bacteria	51.1	BA000030.3
34	*Streptomyces avermitilis* (strain MA-4680)	Bacteria	47.0	NC_003155.4
35	*Streptomyces sioyaensis*	Bacteria	51.4	AF217415.1
36	*Streptomyces sp.* (strain AP77)	Bacteria	43.6	AB127079.1
37	*Trichoderma asperellum*	Fungi	107.9	EU314718.1
38	*Trichoderma viride*	Fungi	85.3	EF176582.1

Supplementary Table 2.

DNA sequence of gene encoding the laminaribiose ABC transporter from *C. thermocellum* after codon usage optimization for yeast (GeneArt GeneOptimizer, Life Technologies).

ATGAAGAAGTTCTTGGCCTTGTTGTTGTCCGTTATCATCGTTTTTTCTTTGACTGCTTG
TGGTGGTAAGAACTCTGGTAACAATTCTAACAACTCCTCCAACAACAACTCCAGTAA
CAATACTGGTGGTAAAAAGATCAAGATCGGTATGGTTACTGATGTTGGTGGTGTTAA
TGACGGTTCCTTTAATCAATCTGCTTGGGAAGGTTTACAAAGAGCCCAAAAAGAATT
GGGTGTTGAAGTTAGATATGCTGAATCTGCTACTGATGCTGATTACGCTCCAAATATT
GAAGCCTTCATTGATGAAGGTTACGACTTGATTATTTGCGTCGGTTATATGTTGGCTG
ATGCTACTAGAAAAGCTGCTGAAGCTAATCCAAATCAAAAGTTCGCCATTATCGATG
ATGCCTCCATTGATTTGCCAAACGTTACTTGTTTGATGTTTGAACAATCCCAAGCCTC
TTACTTGGTTGGTTTGGTTGCTGGTAAAATGACTAAGACTAACAAGGTTGGTTTCGTC
GTTGGTATGGTTTCTCAAACTATGAACGAATTCGGTTACGGTTATTTGGCTGGTGTCA
AAGATGCTAATCCTAACGCTACCATCTTGCAATTCAATGCCAACTCTTTCTCTTCTAC
TGAAACTGGTAAATCTGCTGCTACTACCATGATTACTAATGGTGCCGATGTTATTTTT
CATGCTGCTGGTGGTACTGGTTTGGGTGTTATTGAAGGTTGTAAAGATGCAGGTAAA
TGGGCCATTGGTGTTGATTCTGATCAATCTCCATTGGCCCCAGAAAACATTTTGACAT
CTGCAATGAAGAGAGTTGATAACGCCTGTTTCGATATTGCTAAGGCTGTAAAAGAAG
GTAACGTTAAGCCAGGTATTATCACCTACGATTTGAAATCTGCCGGTGTTGATATTGC
TCCAACTACTACTAATTTGCCTAAAGAAGTTTTGGACTACGTCAATCAAGCCAAGCA
AGATATTATCAACGGTAAGATCACTGTCCCAAAGACTAAGGCTGAATTTGAAGCTAA
GTACGGTAACATCTACGAATTGGATGACG

Supplementary Video Legends

Supplementary Video 1. Direct interaction of (1,3)-β-glucan and (1,4)-β-glucan macrofibrils determines cell wall architecture of epidermal leaf cells in maize.

3D Super-resolution imaging of maize epidermal leaf cell walls co-stained with aniline blue fluorochrome for specific (1,3)-β-glucan detection (blue channel) and pontamine fast scarlet 4B for specific (1,4)-β-glucan detection (red channel) by super-resolution localization microscopy. Video generation with Bitplane Imaris 7.6.1. (Bitplane, Switzerland). Interaction of (1,3)-β-glucan and (1,4)-β-glucan cell wall polymers mainly based on direct macrofibrils interaction.

Supplementary Video 2. Direct interaction of (1,3)-β-glucan and (1,4)-β-glucan macrofibrils determines cell wall architecture of epidermal leaf cells in Miscanthus.

3D Super-resolution imaging of Miscanthus epidermal leaf cell walls co-stained with aniline blue fluorochrome for specific (1,3)-β-glucan detection (blue channel) and pontamine fast scarlet 4B for specific (1,4)-β-glucan detection (red channel) by super-resolution localization microscopy. Video generation with Bitplane Imaris 7.6.1. (Bitplane, Switzerland). Interaction of (1,3)-β-glucan and (1,4)-β-glucan cell wall polymers stronger than in maize and mainly based on direct macrofibrils interaction and additional partial surrounding of (1,4)-β-glucan macrofibrils by (1,3)-β-glucan macrofibrils.

Supplementary Video 3. (1,3)-β-Glucan deposition in the central pith region of bladderwrack blades.

High-resolution imaging of elongated cells within the central pith region of bladderwrack blades stained with aniline blue fluorochrome for specific (1,3)-β-glucan detection by confocal laser-scanning microscopy. Video generation from maximum intensity reconstruction using integral functions of the ZEN 2010 (Zeiss MicroImaging GmbH) operating software. Scattered (1,3)-β-glucan deposition pattern in elongated cells of the central pith region.

Supplementary Video 4. (1,3)-β-Glucan deposition in the cortex and epidermis of bladderwrack blades.

High-resolution imaging of cortex and epidermal cells of bladderwrack blades stained with aniline blue fluorochrome for specific (1,3)-β-glucan detection by confocal laser-scanning microscopy. Video generation from maximum intensity reconstruction using integral functions of the ZEN 2010 (Zeiss MicroImaging GmbH) operating software. Deposition of (1,3)-β-glucan in relatively compact, intracellular layer in cortex and especially epidermis cells.

Supplementary Methods

Heterologous (1,3)-*β*-glucanase expression and purification

Full length cDNAs encoding (1,3)-*β*-glucanases from different organisms (Supplementary Tab. 1) were synthesized with DNA recombination sequences (*attB* sites) at their 5' and 3' ends (GeneArt, Germany) for subsequent utilization with the Gateway cloning technology (Life Technologies, USA). After introduction into the donor vector pDONR221 (Life Technologies) via BP Clonase-mediated recombination, the (1,3)-*β*-glucanase genes were introduced into the *Escherichia coli* expression vector pDEST17 (Life Technologies), providing N-terminal fusion with 6xHis after successful expression, via LR Clonase-mediated recombination. Expression vectors were transformed into *E. coli* strain Rosetta (DE3). *E. coli* cultures were set to D_{600} = 0.5 - 0.8 in Luria-Bertani (LB) bacterial growth media (supplemented with 100 $\mu g \cdot ml^{-1}$ ampicillin and 30 $\mu g \cdot ml^{-1}$ chloramphenicol) and cultivated overnight at 25°C. Heterologous gene expression was induced by adding IPTG (0.5 mM) to the cultures, which were then cultivated overnight at 17°C and 160 rpm to reduce inclusion body formation. For (1,3)-*β*-glucanase purification, *E. coli* cultures were centrifuged and the cell pellets washed with Tris-HCl (20 mM). Cell pellets were then resuspended in native binding buffer (0.2 M NaH_2PO_4, 0.2 M Na_2HPO_4, 29.22 $mg \cdot ml^{-1}$ NaCl) supplemented with 5 $ml \cdot g^{-1}$ pellet imidazole (50 mM, pH 7.4). Lysozyme (0.2 $mg \cdot ml^{-1}$) and PMSF (1 mM) were added, and samples were incubated on ice for 1 h for cell lysis. After sonication, DNA was degraded with the addition of DNase (5 $\mu g \cdot ml^{-1}$) and $MgCl_2$ (10 mM) and an incubation on ice for 15 min. Cell lysates were centrifuged (20 min, 4000 rpm, 4°C) and supernatant containing the His-tagged (1,3)-*β*-glucanases were incubated with Ni-NTA agarose (Qiagen, Germany) in native binding buffer. Washing and elution of His-tagged (1,3)-*β*-glucanases from Ni-NTA agarose followed the manufacturer's instructions (native elution buffer with 500 mM imidazole). For subsequent activity assays, the buffer of the protein samples was exchanged (new buffer conditions: pH 6, 87.7 mM NaH_2PO_4, 12.3 mM Na_2HPO_4) by using Amicon ultra centrifugal filters (Merck Millipore, Germany).

(1,3)-*β*-glucanase activity assay

Purified (1,3)-*β*-glucanases at a final concentration of 100 $\mu g \cdot ml^{-1}$ were incubated overnight in 100 ml activity buffer (pH 6, 87.7 mM NaH_2PO_4, 12.3 mM Na_2HPO_4) containing 500 $\mu g \cdot ml^{-1}$ (1,3)-*β*-glucan from *E. gracilis* or *Laminaria digitata* (Sigma-Aldrich) as substrate at 37°C. Commercially available (1,3)-*β*-glucanase from *T. reesei* (Sigma-Aldrich) served as reference.

Release of glucose due to (1,3)-*β*-glucan hydrolysis was used to determine (1,3)-*β*-glucanase activity. Quantification of glucose followed the description from Stitt *et al.*[1] in photometrical assays in 96-well plates.
To determine the optimal temperature of (1,3)-*β*-glucanase activity, enzyme samples were incubated overnight in the activity buffer as described above, however, at temperatures ranging from 25°C to 55°C. pH dependency of (1,3)-*β*-glucanase activity was measured from overnight incubations at 37°C using an acetate buffer system in the range from pH 3 to pH 5 and a phosphate buffer system in the range from pH 6 to pH 9 [2].

Laminaribiose transporter yeast strain generation

To improve utilization of the (1,3)-*β*-glucan hydrolysis products laminaribiose and laminaritriose, the yeast (*S. cerevisiae*) strain CEN.PK113-13D (CEN, *MATα MAL2-8^c SUC2 ura3-52*) was transformed with an expression vector carrying the bacterial laminaribiose ABC transporter (LBT) from *C. thermocellum*[3]. For expression vector generation, the full length cDNA encoding LBT was codon optimized for yeast expression (sequence see Supplementary Tab. 2) and synthesized with DNA recombination sequences (*attB* sites) at their 5' and 3' ends (ShineGene, China) for subsequent utilization with the Gateway cloning technology. After introduction into the donor vector pDONR221 via BP Clonase-mediated recombination, the *LBT* gene was introduced into the yeast expression vector pYES-DEST52-GAP, providing C-terminal fusion with 6xHis after successful expression, via LR Clonase-mediated recombination. The vector pYES-DEST52-GAP was generated by replacing the inducible galactokinase (GAL1) promoter of the expression vector pYES-DEST52 (Life technologies) by the constitutive glyceraldehyde-3-phopshate dehydrogenase (GAP) promoter of the *Pichia pastoris* expression vector pGAPZ (Life technologies). The GAP promoter sequence was amplified via PCR reaction using primers that provide endonuclease recognition sites at the 5'-end (*Spe*I, GAP-5'Spe: 5'-CGAAAACACTAGTTAAGGGAT TTTGGTC) and the 3'-end (*Acc*III, GAP-3'Acc: 5'-GCTCACCGTCTTTCATTGTCCGGA) of the amplified PCR fragment. After *Spe*I/*Acc*III endonuclease treatment of the GAP fragment, it was cloned into the likewise prepared yeast expression vector pYES-DEST52 via sticky-end ligation.
The yeast strain CEN was transformed with the respective expression vector containing the *LBT* gene under the control of the constitutive GAP promoter using the lithium-acetate method for yeast transformation[4]. Based on its slightly better growth characteristics on glucose,

laminaribiose, and laminaritriose (Fig. 2b), the transformed CEN strain CEN+LBT #2 was used in all subsequent growth assays and biomass fermentations and referred to as “CEN+LBT”.

Yeast growth assay

Overnight cultures (28°C, 150 rpm) of the CEN and CEN+LBT yeast strains were cultivated in 5 ml SD liquid medium (6.7 $g \cdot l^{-1}$ yeast nitrogen base w/o amino acids (BD, USA), 0.78 $g \cdot l^{-1}$ dropout powder w/o uracil (Clontech, USA), 20 $g \cdot l^{-1}$ glucose, and with (CEN) or without (CEN+LBT) 40 $mg \cdot l^{-1}$ uracil (Sigma-Aldrich), pH 5,8). Yeast cultures were centrifuged and pellets washed with H_2O. After additional centrifugation, the pellet was resuspended in 10 ml H_2O. Growth assays were performed in 96-well plates in 200 µl minimal medium (SD liquid medium without glucose) with a carbon source concentration of 1 $g \cdot l^{-1}$. Yeast starting concentrations were set to D_{600} = 0.01. Carbon sources tested were glucose, (1,3)-*β*-glucan from *E. gracilis*, laminaribiose (this and all following oligosaccharides from Megazyme), laminaritriose, laminaritetraose, cellobiose, cellotriose, cellotetraose, glucotriose (I) (*β*-D-Glc-(1→3)-*β*-D-Glc-(1→4)-D-Glc), glucotriose (II) (*β*-D-Glc-(1→4)-*β*-D-Glc-(1→3)-D-Glc), glucotetraose (I) (*β*-D-Glc-(1→3)-[*β*-D-Glc-(1→4)]$_2$-D-Glc), glucotetraose (II) ([*β*-D-Glc-(1→4)]$_2$-*β*-D-Glc-(1→3)$_2$-D-Glc), and), glucotetraose (III) (*β*-D-Glc-(1→4)-*β*-D-Glc-(1→3)$_2$-*β*-D-Glc-(1→4)-D-Glc). 96-well plates with growth assays were incubated at 28°C and 150 rpm for 72 h. D_{600} was measured every 24 h in a microplate reader (BioTek).

Western blot analysis

Expression and size of the six (1,3)-*β*-glucanases #11, #15, #21, #34, #36, and #37 (Supplementary Tab. 1), which showed highest enzymatic activity (Supplementary Figs. 3 and 6), and of LBT in the yeast strains CEN+LBT #1 and #2 were confirmed via Western blot analysis. Proteins were separated on an SDS-PAGE and transferred on nitrocellulose membranes using transfer buffer (Thermo Scientific, USA) in a Trans-Blot electrophoretic transfer cell (Biorad, USA). After protein transfer, membranes were washed and additionally treated with the Pierce Western blot signal enhancer kit (Thermo Scientific) according to the manufacturer’s instructions. Blocking of membranes was done in Tris-buffered saline with Tween 20 (TBST) buffer containing 5 % skimmed milk powder. Membranes were then incubated overnight at 8°C with an anti-His antibody (Roche, Germany) as primary antibody in TBST with 1 % skimmed milk powder. A secondary, horseradish peroxidase (HRP)-conjugated anti-mouse IgG antibody

(Promega, Germany) was used according to the manufacturer's instructions. Hybridization of the antibodies with the respective proteins was detected by chemiluminescence using the SuperSignal West Pico substrate (Thermo Scientific). Identity of tested proteins was additionally verified by mass spectrometry.

Chemical hydrolysis of *β*-glucans

5 mg of (1,3)-*β*-glucan from *E. gracilis*, the (1,4)-*β*-glucan cellulose, and the mixed-linkage (1,3;1,4)-*β*-glucan from barley (all glucans from Sigma-Aldrich) were treated with 1 ml of 1 M trifluoroacetic acid or 1 M sulfuric acid for 3 h at 105°C. The amount of released glucose was quantified with a refractive index detector as described above.

Miscanthus transformation

For callus induction, young, immature inflorescences from Miscanthus were surface sterilized with sodium hypochlorite (2 %) and then with ethanol (70 %), each for 5 min. After rinsing with H_2O, inflorescences were cut into 1 cm pieces and transferred on callus induction medium (CIM) (30 $g \cdot l^{-1}$ sucrose, 4.3 $g \cdot l^{-1}$ Murashige and Skoog Basal Salt Mixture (MS), 2 $g \cdot l^{-1}$ phytagel, 112 $mg \cdot l^{-1}$ vitamin B5, $MgCl_2$ (final concentration 3.69 mM), 2,4-dichlorophenoxyacetic acid (13.57 µM), 6-benzylaminopurine (0.44 µM), L-proline (24.33 mM), pH 5.8) and incubated in the dark at 28°C for 3 – 4 weeks. For Agrobacteria-mediated callus transformation, calli were incubated with infiltration medium (IM) (10 $g \cdot l^{-1}$ sucrose, 4.3 $g \cdot l^{-1}$ MS, 10 $g \cdot l^{-1}$ mannitol, acetosyringone (final concentration 2.29 µM), pH 5.5) containing Agrobacteria strains (carrying vector constructs as described above) on CIM plates for 30 min. After incubation, Agrobacterium suspension was removed. Calli were dried on filter paper, transferred onto filter paper soaked with IM, and incubated in petri dishes at room temperature in the dark for 7 d. To remove Agrobacteria after co-incubation, calli were first rinsed with H_2O and then with a ticarcillin disodium mixture solution (500 $mg \cdot ml^{-1}$, Duchefa, Netherlands). After overnight incubation in the ticarcillin disodium mixture solution, calli were rinsed with water, dried on filter paper, and transferred on selection medium (30 $g \cdot l^{-1}$ sucrose, 4.3 $g \cdot l^{-1}$ MS, 2 $g \cdot l^{-1}$ phytagel, 112 $mg \cdot l^{-1}$ vitamin B5, 224 $mg \cdot l^{-1}$ ticarcillin disodium mixture, 50 $mg \cdot l^{-1}$ hygromycin, $MgCl_2$ (final concentration 3.69 mM), 2,4-dichlorophenoxyacetic acid (13.57 µM), 6-benzylaminopurine (0.44 µM), L-proline (173.76 µM), glycine (266.67 µM), L-asparagine monohydrate (66.6 µM), pH 5.8). After incubation in the dark at 28°C for 2 – 3 weeks, surviving calli were transferred on regeneration medium (20 $g \cdot l^{-1}$ sucrose, 4.3 $g \cdot l^{-1}$ MS, 10 $g \cdot l^{-1}$ mannitol, 2 $g \cdot l^{-1}$ phytagel, 112 $mg \cdot l^{-1}$

vitamin B5, 124.8 mg·l^{-1} ticarcillin disodium mixture, 1-naphthaleneacetic acid (final concentration 2.69 µM), kinetin (2.32 µM), pH 5.8) and incubated at 16 h of light at 25°C. Regenerated plants (approximate size 5 mm) were then transferred on germination medium (30 g·l^{-1} sucrose, 4.3 g·l^{-1} MS, 14 g·l^{-1} activated charcoal, 2 g·l^{-1} phytagel, 112 mg·l^{-1} vitamin B5, 124.8 mg·l^{-1} ticarcillin disodium mixture, 30 mg·l^{-1} hygromycin, 1-naphthaleneacetic acid (final concentration 0.54 µM), 6-benzylaminopurine (8.88 µM), L-glutamine (342.14 µM), pH 5.8) to induce root formation at 16 h of light at 25°C. Plants with roots were transferred on a soil/sand mixture (1/1 (v/v)) for continuous growth. If not indicated otherwise, all chemicals purchased at Sigma-Aldrich.

Gene expression analysis

To confirm expression of *PMR4-GFP* and *GFP* in transformed Miscanthus lines, RNA was isolated from wild-type and the generated lines *35S:GFP* and *35S:PMR4-GFP* #1 and #2 lines using peqGOLD TriFast (Peqlab, Germany) according to the manufacturer's instructions. For complementary DNA (cDNA) synthesis, the Maxima First Strand cDNA Synthesis Kit (Thermo Scientific, USA) was used following the instructions of the manufacturer. The expression of *Actin* (Primer, 5'Act: 5'-ATGCACCAAGAGCTGTCTTC and 3'Act: 5'-GCTGGGCATTCAAAGGTTTC; primer sequences derived from *Miscanthus sinensis Actin* sequence (GenBank Acc. No. JN983213.1)) was used as reference for the expression of *PMR-GFP* (5'PMR4: 5'-ATCCAATACGCCCGTGAC and 3'GFP: GTGGCGGATCTTG AAGTTCAC) and *GFP* (5'GFP: 5'-ACGACGGCAACTACAAGAC and 3'GFP) in PCR reactions using the Q5 High-Fidelity DNA Polymerase (New England Biolabs, USA). Identity of amplified PCR products was confirmed by DNA sequencing.

1 Stitt, M., Lilley, R. M., Gerhardt, R. & Heldt, H. W. Metabolite levels in specific cells and subcellular compartments of plant leaves. *Methods Enzymol.* **174**, 518-552 (1989).

2 in *Lab FAQs - Find a Quick Solution* (eds U.W. Hoffmann-Rohrer & B. Kruchen) Ch. 5.1. Buffers, 153-166 (Roche Diagnostics GmbH, 2011).

3 Nataf, Y. *et al.* Cellodextrin and laminaribiose ABC transporters in *Clostridium thermocellum*. *J. Bacteriol.* **191**, 203-209 (2009).

4 Gietz, D., St Jean, A., Woods, R. A. & Schiestl, R. H. Improved method for high efficiency transformation of intact yeast cells. *Nucleic Acids Res.* **20**, 1425 (1992).
